# 1856. Risk factors for mortality amongst hospitalised patients with *Staphylococcus aureus* bacteraemia – a prospective cohort study

**DOI:** 10.1093/ofid/ofac492.1485

**Published:** 2022-12-15

**Authors:** Jinghao Nicholas Ngiam, Haziq Nasir, Yin Mo, Louis Yi-Ann Chai, Sophia Archuleta, Dale Fisher, Paul Anantharajah Tambyah

**Affiliations:** National University Health System, Singapore, Not Applicable, Singapore; National University Health System Singapore, Singapore, Not Applicable, Singapore; National University Health System, Singapore, Not Applicable, Singapore; National University Health System, Singapore, Not Applicable, Singapore; National University Health System Singapore, Singapore, Not Applicable, Singapore; National University Health System Singapore, Singapore, Not Applicable, Singapore; National University Health System, Singapore, Not Applicable, Singapore

## Abstract

**Background:**

*Staphylococcus aureus* bacteraemia (SAB) remains a common infection with significant mortality, ranging from 20 to 40%. Understanding the risk factors for adverse outcomes in this infection will better prognosticate and guide patient management and disposition.

**Methods:**

We prospectively recruited and examined 634 consecutive patients with laboratory-confirmed SAB. They were characterised in terms of baseline demographics, clinical profile, and laboratory findings at the time of SAB diagnosis. Sensitivity testing was carried out to identify patients who had methicillin-resistant (MRSA) or methicillin-sensitive *Staphylococcus aureus* (MSSA) infection. All patients were followed up prospectively for clinical outcomes including in-hospital mortality, and sites of metastatic infection. Univariate and multivariable logistic regression was used to identify risk factors associated with mortality in hospitalised patients with SAB.

**Results:**

The overall mortality was 5.8% (n=37). There were no significant differences in gender, but patients who died tended to be 11 years older on average (69.6±14.9 years vs 58.1±18.2 years, p< 0.001). Diabetes mellitus, prior history of structural heart disease, haemodialysis and recent surgery had not been significantly associated with mortality in our cohort. Amongst the patients who died, MRSA infection was significantly more common (45.9% vs 24.8%, p=0.004). On multivariable logistic regression, older age (adjusted OR 1.05, 95%CI 1.02-1.07) and MRSA infection (adjusted OR 2.04, 95%CI 1.01 – 4.09) remained independently associated with mortality. We did not demonstrate an association with prolonged fever or bacteraemia and in-hospital mortality.

**Figure:**
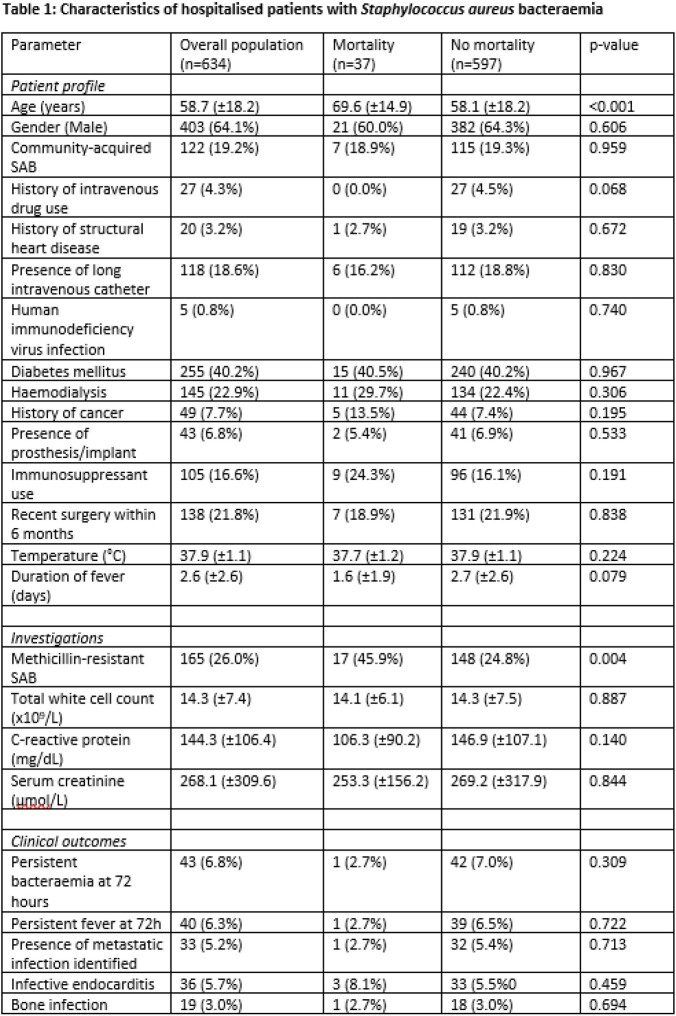

**Figure:**
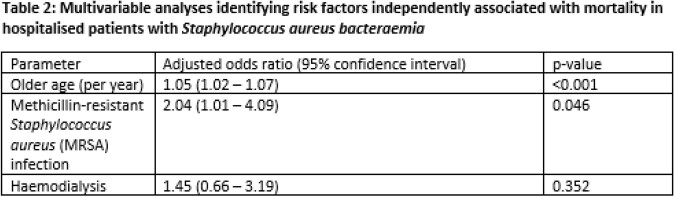

**Conclusion:**

Amongst hospitalised patients with SAB, the mortality was low (5.8%). Older age and MRSA infection were the most important risk factors identified in association with mortality.

**Disclosures:**

**All Authors**: No reported disclosures.

